# Monitoring SARS-CoV-2 genetic variability: A post-market surveillance workflow for combined bioinformatic and laboratory evaluation of commercial RT-PCR assay performance

**DOI:** 10.1371/journal.pone.0294271

**Published:** 2024-01-12

**Authors:** Barbara Kosińska-Selbi, Justyna Kowalczyk, Jagoda Pierscińska, Jarosław Wełeszczuk, Luis Peñarrubia, Benjamin Turner, Josep Pareja, Roberto Porco, Rubi Diaz-Hernandez, Martí Juanola-Falgarona, Melisa Rey, Davide Manissero, Anna Blacha

**Affiliations:** 1 QIAGEN Wrocław Sp. z o.o., Wrocław, Poland; 2 QIAGEN (Previously STAT-Dx Life S.L.), Barcelona, Spain; 3 QIAGEN Digital Insights, Aarhus, Denmark; 4 QIAGEN Manchester Ltd, CityLabs, Manchester, United Kingdom; University of Bologna / Romagna Local Health Authority, ITALY

## Abstract

**Objective:**

The speed at which Severe Acute Respiratory Syndrome Coronavirus-2 (SARS-CoV-2) is mutating has made it necessary to frequently assess how these genomic changes impact the performance of diagnostic real-time polymerase chain reaction (RT-PCR) assays. Herein, we describe a generic three-step workflow to assess the effect of genomic mutations on inclusivity and sensitivity of RT-PCR assays.

**Methods:**

Sequences collected from the Global Initiative on Sharing All Influenza Data (GISAID) were mapped to a SARS-CoV-2 reference genome to evaluate the position and prevalence of mismatches in the oligonucleotide-binding sites of the QIAstat-Dx, an RT-PCR panel designed to detect SARS-CoV-2. The frequency of mutations and their impact on melting temperature were assessed, and sequences flagged by risk-based criteria were examined *in vitro*.

**Results:**

Out of 8,900,393 SARS-CoV-2 genome sequences analyzed, only 173 (0.0019%) genomes contained potentially critical mutations for the QIAstat-Dx; follow-up *in-vitro* testing confirmed no impact on the assays’ performance.

**Conclusions:**

The current study demonstrates that SARS-CoV-2 genetic variants do not affect the performance of the QIAstat-Dx device. It is recommended that manufacturers incorporate this workflow into obligatory post-marketing surveillance activities, as this approach could potentially enhance genetic monitoring of their product.

## Introduction

Pathogens continuously adapt to pressures imposed by numerous factors, such as host immune systems and therapeutic agents that target the infections they cause. These adaptive forces can lead to genomic changes (mutations or rearrangements) in the pathogen, significantly affecting its transmission potential or virulence [1]. It is crucial to recognize that these genomic alterations may negatively impact the complementarity and primer annealing capacity of diagnostic RT-PCR assays [2]. In the context of a specific RT-PCR assay setup, the ability of primers to bind to the target sequence at a given temperature (annealing) plays a vital role in determining the specificity and efficiency of the amplification process. Genetic variations can cause mismatches between primer sequences and the target sequence which, in turn, compromise the stability of the primer–target complex. Consequently, the probability of complex formation decreases, subsequently affecting the assay’s sensitivity and increasing the likelihood of false-negative results [3].

Alluding to this, since the original outbreak of the Severe Acute Respiratory Syndrome Coronavirus-2 (SARS-CoV-2) in Wuhan, China, in late 2019, evolutionary pressures have contributed to the constant mutation and recombination of viral genetic material over time, generating novel SARS-CoV-2 variants, such as the Delta and Omicron lineages, which have considerably extended the global Coronavirus Disease 2019 (COVID-19) pandemics [4]. However, during the preliminary stages of the outbreak, the stark uncertainty about genomic evolution of SARS-CoV-2 sparked insightful development of RT-PCR assays capable of detecting multiple regions of the virus’ original genome, which could aid in reducing test susceptibility to genetic variation. For example, the panel proposed by the Charité-Universitätsmedizin Berlin Institute of Virology and endorsed by the World Health Organization (WHO) was design to target the RdRp, E and N genes of the SARS-CoV-2 Genome. Another panel, proposed by CDC, contained one additional assay (called N3, also targeting N gene) designed for universal detection of SARS-like coronaviruses [5].

One strategy employed by manufacturers of diagnostic assays, which adheres to *in-vitro* diagnostic regulatory stipulations set forth by authoritative entities, involves the performance of post-market surveillance (PMS) activities. A PMS system includes the diligent and methodical observation of a diagnostic assay’s safety and efficacy by acquiring and examining publicly accessible data pertinent to its application and indications for use [6]. Manufacturers are obligated to assess the potential impact of pathogen genetic variation on their diagnostic assays’ sensitivity. Consequently, PMS initiatives, specifically designed to routinely evaluate diagnostic assay performance, must be developed and implemented by manufacturers in compliance with these requirements.

To address the constant rate of genomic variability in SARS-CoV-2, regulatory agencies have released guidelines for COVID-19 diagnostic test manufacturers, mandating the implementation of PMS activities [7, 8]. This requires routine monitoring of viral mutations that may impact test performance to detect and correct any possible test limitations in the product labeling, as well as mitigating the potential for false-negative and false-positive results [7, 8]. Hence, the objective of the current study was to assess the impact of SARS-CoV-2 genetic variability on the sensitivity and inclusivity of the QIAstat-Dx SARS-CoV-2 Panel and the QIAstat-Dx SARS-CoV-2/Flu A/B/RSV Panel (QIAstat-Dx; Qiagen^®^ GmbH, Hilden, Germany)—both of which are RT-PCR-based assays—using a generic, three-step workflow that may provide a basis for PMS systems to evolve and adapt to future pandemics and pathogen genetic variations.

As an overview, the first step in the proposed three-step workflow involves the collection of sequences from publicly accessible databases. The second step is an *in-silico* evaluation of publicly recorded genetic variability of any pathogen’s genome on the specific region complementary to the target oligonucleotides of an assay, including an analysis of positional mismatches and thermodynamic calculations to assess the impact of mismatches on annealing temperature and RT-PCR reaction efficiency. These factors, in addition to the frequency by which the mismatch occurs, are used to determine the criticality of the mutations. The third step begins with a formulative decision to submit potentially critical mismatches for *in-vitro* testing, based on the criticality of mismatches (position and its impact on annealing temperature) plus the relative frequency of the mismatched sequences within the analyzed time period as well as between consecutively analyzed time periods. The result from this formulative decision-making process would enable manufacturers to objectively determine whether a mismatch impacts the sensitivity of an RT-PCR assay, thereby allowing them to act on any negative outcomes accordingly.

## Materials and methods

### Technical analysis workflow

The proposed workflow originates from a previously described procedure [5, 9], modified to include the following three steps in order to optimize the analyses:

1. Sequence collection
  - A set of sequences uploaded to the publicly available databases during a given period were obtained as FASTA-formatted input. Sequences were filtered based on the length (>26,000 bp) and content of missing nucleotides.
2. Mapping and false negative assessment
  - A high-sensitivity mapping algorithm implemented in the *Geneious Prime* software (Biomatters, Ltd., Auckland, New Zealand) (https://manual.geneious.com/en/latest/) was used to map all of the sequences in the dataset obtained in step 1, to the reference sequence with the annotated oligonucleotides of the QIAstat-Dx devices. Afterwards, custom Python scripts were implemented to automatically process the assembly of FASTA-formatted files. This automated process identifies the mismatches within oligonucleotide-binding regions (*E* and *RdRp*). In addition, a custom R script was used to investigate whether both targets are potentially at risk based on the same genome IDs simultaneously.
  - Appropriate primer–target sequence melting temperatures were estimated for the sequences presenting one or more mismatches using a custom script based on the *rmelting* package version 1.8.0 from the Bioconductor Software Packages version 3.16 (www.bioconductor.org). Specific concentrations of salts, oligos, and nucleotides were adjusted based on QIAstat-Dx cartridge configuration.
  - An overall criticality assessment was performed according to **Table 1**.
  - A frequency analysis was performed to assess if:
    ■ A critical mismatch or mismatch pattern was present in more than 100 sequences throughout the analysis period.
    ■ There was a four-fold increase in frequency of the specific mismatch or mismatch pattern in the given dataset (corresponding to a specific period) relative to the previous analysis.
3. *In-vitro* testing of the critical sequences
  - Mutations and/or patterns of mutations that were assessed as potentially critical according to the **Table 1** criteria and met both criteria for frequency, as described in step 2, were selected for *in-vitro* testing using an artificial double-stranded deoxyribonucleic acid (dsDNA) sequence. This dsDNA molecule, covering the original mismatch or mismatches pattern, was ordered from the supplier, diluted around the limit of detection concentration, and tested using the commercial assay to assess the potential impact on sensitivity.

**Table 1:** Decision criteria for *in-vitro* test.

Oligonucleotide	Location of mismatches	Acceptance criteria	Impact	Risk assessment
**Primer** (applicable to forward or reverse primer)	3’ end	One or more new mismatches within the last three nucleotides	Critical to detection	High
	Anywhere in the primer sequence	If three or more new mismatches	Critical to detection	High
		If two mismatches, assess the estimated T_m_	Critical to detection if T_m_ of the primer is markedly below (at least 2°C) the annealing temperature of the thermal profile	High
**TaqMan probe**	5’ end	One or more new mismatches within the last three nucleotides	Critical to detection	High
	Anywhere in the probe sequence	Three or more new mismatches	Critical to detection	High
		If two mismatches, assess the estimated T_m_	Critical to detection if T_m_ of the probe is more than 2°C lower than the T_m_ of the primer binding in the same direction	High

T_m_, melting temperature.

Ethical approval was not required for this work as it consisted of routine surveillance activities.

### Assessment of the SARS-CoV-2 assays on the QIAstat-Dx panels

The SARS-CoV-2 assay component of the QIAstat-Dx devices combines two RT-PCR assays that target *E* and the ribonucleic acid (RNA)-dependent RNA polymerase (*RdRp*) segment of the open reading frame 1ab (ORF1ab) region of the SARS-CoV-2 genome [10]. Below is the combined analysis of these assays as implemented according to the described workflow.

#### Sequence collection

The complete set of known SARS-CoV-2 genomes published in the Global Initiative on Sharing All Influenza Data (GISAID) EpiCoV database [11] were processed periodically (minimum once a month) using a custom script filtering genome length of ≥26,000 bp (to avoid analyzing incomplete genomes). Sequences with multiple segments that had 290 or more ambiguous nucleotides (i.e., stretches of NNNs) were excluded from analysis. In the early stage of the analysis, sequences from the National Center for Biotechnology Information nucleotide database (GenBank; https://www.ncbi.nlm.nih.gov/genbank/) were also used to achieve a coverage of genetic patterns.

#### Mismatch analysis

**Fig 1:**
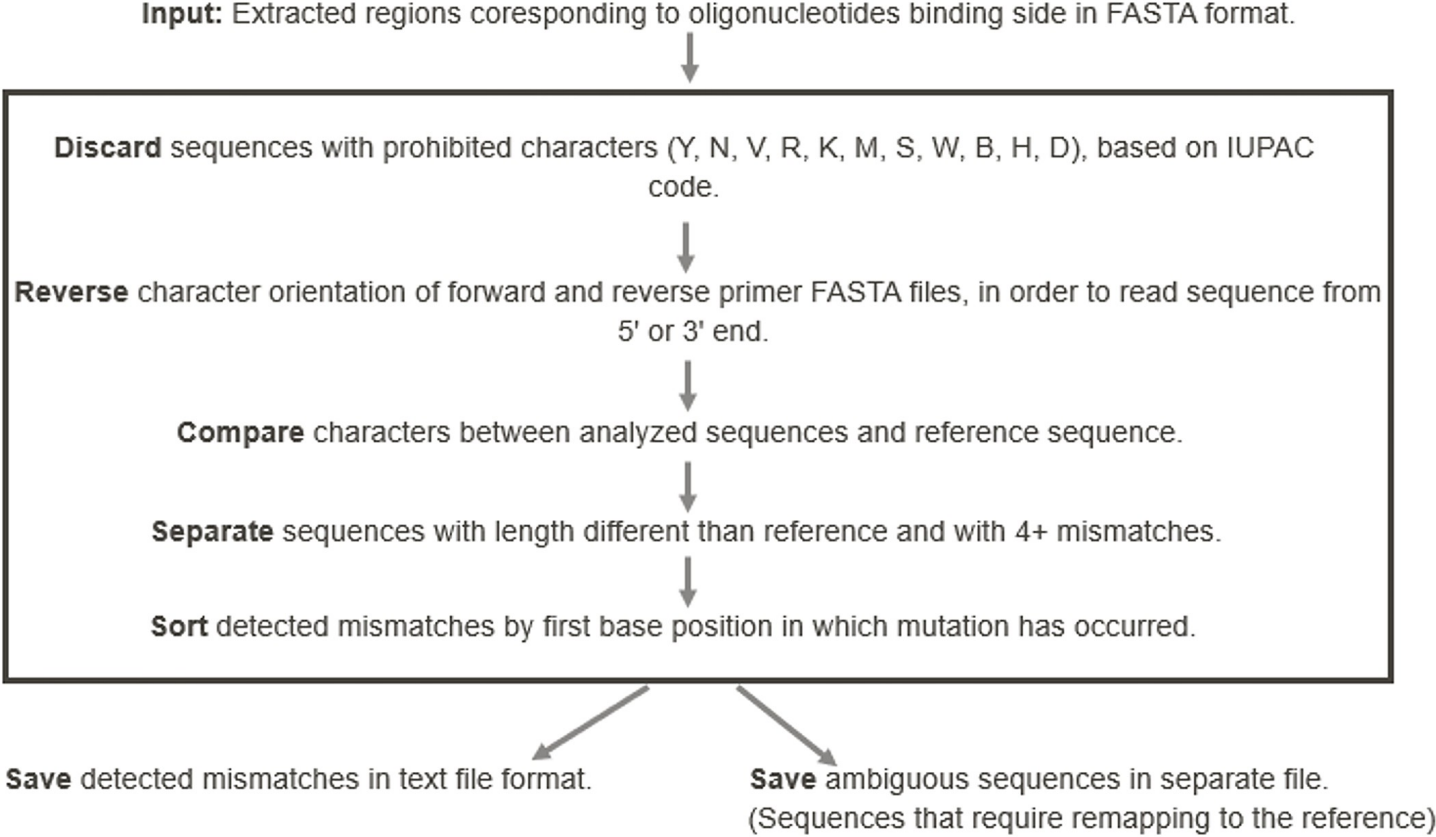
Scheme of operations for detecting mutations from regions extracted from the mapping.

Filtered sequences were uploaded to the *Geneious Prime* software and mapped to a SARS-CoV-2 reference genome (GenBank accession no. NC_045512.2). Regions corresponding to the target’s oligonucleotide-binding sites were extracted, and those lacking mutations in these binding sites were considered completely detected and consequently excluded from analysis. Subsequently, mismatch patterns were characterized from the extracted regions using a custom written python script. Observed mutation patterns were sorted based on the number of mismatched nucleotides found in the pattern. As a confirmation step, sequences which were shown to have more than four mismatched nucleotides in the pattern, were re-analyzed by performing an individual mapping for confirmation. This is because it was noticed that, when mapping multiple sequences to a reference sequence, some of the multiple mutations resulted in a false alignment. Therefore, this additional step allows to optimize the process algorithm and to mitigate possible errors. Potentially critical mutations were then assessed in follow-up *in-vitro* testing as described in step 3. The script’s operation protocol is shown in Fig 1.

#### Detection of mutation in both targeted regions

Based on results obtained from the mismatch detection step, an additional analysis was performed to identify combinations of critical and/or noncritical mutation patterns within both QIAstat-Dx panels targets amplicons simultaneously. This included all combinations of potentially critical and noncritical mutations among both amplicons. The purpose of this analysis was to detect whether critical changes occur in both QIAstat-Dx targets based on the same genome IDs. If critical changes occurred in both targets, this could potentially lead to a substantial risk in impaired pathogen detection. As a mitigation, a custom R script was included to analyze mutations found in both targeted genes among genomes.

#### Analysis of VOCs and VOIs

Additionally, owing to specific regulatory requests, the same analysis was performed specifically on the SARS-CoV-2 variants classified as Variants of Concern (VOCs) and Variants of Interest (VOIs). These are a subset of the previously analyzed sequences. Classification of VOCs and VOIs was obtained from official sources from the World Health Organization, the US Centers for Disease Control and Prevention, the European Centre for Disease Prevention and Control, and the UK Medicines and Healthcare products Regulatory Agency [12–16] and the global report on the Phylogenetic Assignment of Named Global Outbreak (PANGO) lineage website [17]; The list of variants analyzed during the implementation of the PMS workflow are provided in **S1 Table,** including major sublineages within the Alpha, Beta, Gamma, Eta, B.1.1.318, Delta, B.1.617.3, Mu, and Omicron variants.

#### *In-vitro* testing

*In-vitro* testing was performed using synthetic dsDNA sequences (gBlocks™ Gene Fragments, Integrated DNA Technologies, Coralville, IA, USA) to assess the impact of the flagged sequences on the assay sensitivity. The gBlocks™ were diluted at three times (3×) the limit of detection (LoD) using universal transport medium. For the *in vitro* testing, 1500 copies/ml were used, which corresponds to 500 copies/ml per one LoD concentration. A minimum of 20 replicates were tested in accordance with the US Food and Drug Administration recommendations for SARS-CoV-2 assay manufacturers [7]. To assess the sensitivity of the SARS-CoV-2 assays on the QIAstat-Dx panels, two gBlocks™ sequences were combined in each specimen, representing the two genomic regions targeted by the devices. In all cases, the flagged sequence (containing the mismatches) was present in one of the two gBlocks™, whereas the second gBlock™ corresponded to the one in the reference genome. Reference genome gBlocks™ were also run at the same concentration as a positive control. Acceptance criteria were established at positive detection ≥95% with a 95% confidence interval (CI) >80%. Practically speaking, two or more false-negative results imply failure of the acceptance criteria. In case one false-negative result was observed, additional 20 runs were performed to establish whether that specific sequence could meet the CI acceptance criteria.

## Results

### Sequences collected

The analyzed data included 8,900,393 genome sequences obtained between January 1^st^, 2020, and July 6^th^, 2022, with 8,643,408 and 256,985 sequences obtained from GISAID and partially from GenBank (including data until May 2^nd^, 2021) databases, respectively. The GISAID data was filtered out from 11,628,150 entries with completed information about the collection date (http://doi.org/10.55876/gis8.230206vz) available between January 1^st^, 2020, and July 6^th^, 2022. This sequence collection covered all relevant SARS-CoV-2 lineages, including B.1.1.7 (Alpha), B.1.351 (Beta), P.1 (Gamma), B.1.525 (Eta), B.1.1.318, B.1.617.2 (Delta), XD (Deltacron), B.1.617.3, B.1.621 (Mu), B.1.529 (Omicron), C.37 (Lambda), and B.1.640 [12–16, 19], yielding a total of 505 variants/subvariants (**S1 Table**).

### Mismatch detection and sensitivity analysis

Of 8,900,393 genomes analyzed, 182,801 (2.05%) contained mismatches in noncritical positions and 25,750 (0.29%) contained mutations in potentially critical positions within the QIAstat-Dx oligonucleotide-binding sites. Prevalence of mismatches, and potentially critical and noncritical mutations for both amplicons are shown in **Table 2**. Among the 10 most prevalent critical mismatches (**Table 3**), we detected mutations characteristic of the AY.4.4, AY.22, and AY.102.2 Delta sublineages. Specifically, AY.4.4 and AY.22 sublineages contained mismatches in the oligonucleotide-binding region of the ORF1ab amplicon (reverse primer), while AY.102 sublineage contained mismatches in the oligonucleotide-binding site of the *E* amplicon (probe sequence). Overall, the most prevalent critical pattern was observed for the reverse primer of the *E* target and its frequency was below 0.5% (**Table 3**). **S2 Table** lists all detected mismatches with estimated melting temperatures, the number of mismatched nucleotides and genomes, frequency, and criticality of the mismatch.

**Table 2:** Results for the mismatch analysis.

	Sequences with any mismatches^a^	Sequences with noncritical mismatches	Sequences with critical mismatches
***RdRp* target: Total number (%)**	118,400 (1.33)	107,253 (1.205)	12,021 (0.14)
***E* target: Total number (%)**	85,761 (0.96)	76,923 (0.86)	13,902 (0.16)
**Both targets:**^**b**^ **Total number (%)**	202,292 (2.27)	182,801 (2.05)	25,750 (0.29)

^a^The number of sequences with any mismatches is lower than the sum of sequences with noncritical mismatches and sequences with critical mismatches as the same sequence can contain both: potentially critical and noncritical mismatches in different oligos of the same amplicon.

^b^Numbers for both targets are lower than the simple sum of numbers for *E* and *RdRp* amplicons. Sequences that have mutations in both amplicons have been considered in the calculations.

**Table 3:** Ten most prevalent critical single bp mismatches.

Target gene	Oligonucleotide	Occurrence in specific	Number of sequences (%)
		SARS-CoV-2 variant	
*E*	Reverse primer	N/A	9,895 (0.111)
*RdRp*	Reverse primer	AY.4.4 and AY.22	7,690 (0.086)
*E*	Forward primer	N/A	2,226 (0.025)
*RdRp*	Reverse primer	N/A	2,061 (0.023)
*RdRp*	Forward primer	N/A	1,513 (0.017)
*E*	Forward primer	N/A	1,104 (0.012)
*RdRp*	Reverse primer	N/A	1,024 (0.012)
*E*	Fluorescent probe	AY.102.2 sublineage of Delta	814 (0.009)
*RdRp*	Fluorescent probe	N/A	591 (0.007)
*E*	Reverse primer	N/A	504 (0.006)

N/A, not applicable; SARS-CoV-2, severe acute respiratory syndrome coronavirus-2

Eight sequences were flagged to be tested *in vitro*. All sequences yielded a 20/20 expected detection result, except for sequence n3 (**Table 4** and **S3 Table**) that yielded one negative result, which triggered the testing of 20 additional runs that confirmed a total detection of >95% with a 95% CI >80% as per the established acceptance criteria.

**Table 4:** Summary of in-vitro results.

Target	Oligonucleotide	Number of mutation patterns tested	Detection rate at 3× LoD	Conclusion
*E*	Forward primer	1	20/20	No loss of detection sensitivity observed
	Fluorescence probe	1	20/20	
	Reverse primer	2	39/40, 20/20	
*RdRp*	Forward primer	1	20/20	
	Fluorescence probe	0	N/A	
	Reverse primer	3	20/20, 20/20, 20/20	

LoD, level of detection; N/A, not applicable

### Detection of mutation in both targets

Mismatches observed in both target genes were detected in 1,869 (0.021%) genomes, with 1,375 (0.015%) of them classified as noncritical mismatches (**Table 5**). Only 173 (0.002%) genomes contained mismatches in critical positions within an oligonucleotide-binding site of both target amplicons (**S4 Table**). Interestingly, 246 (0.003%) genomes had critical mutations for the *E* target and noncritical mutations for *RdRp*, whereas 136 (0.002%) genomes presented the opposite pattern (**Table 5**). However, because of the small number of sequences containing any of these patterns, these were not flagged for *in-vitro* testing according to the established criteria.

**Table 5:** Detection of genomes showing mutations in both targets (E and RdRp).

	Number of genomes with mutations in *RdRp* and *E* targets	Frequency (%)
Mismatches in both amplicons with any expected impact	1,869	0.021
Only noncritical mismatches	1,375	0.015
Only critical mismatches	173	0.002
With a critical mismatch for *E* and noncritical mismatch for *RdRp*	246	0.003
With a critical mismatch for *RdRp* and noncritical mismatch for *E*	136	0.002

## Discussion

### Performance of the SARS-CoV-2 panels

IVD tests are a crucial tool for healthcare providers to accurately diagnose, prescribe treatment, and monitor patient status. Owing to their key role in public health and to ensure patient safety, commercial assays need to be developed under strict quality standards (ISO 13485). As such, regulatory authorities worldwide review and approve medical devices on a continuous basis to ensure these can be used in clinical settings in an accurate, reliable, and safe manner [7, 8, 18]. Regular PMS activities are one way regulatory authorities assess device performance once it reaches the market. To that end, the current study investigated the clinical performance of SARS-CoV-2 assays included in the QIAstat-Dx devices and assessed their ability to detect the SARS-CoV-2 genome despite rapid mutation of the virus since early 2020. The practical application of the described PMS workflow demonstrated that an extremely small number of potentially critical mutations were detected that could impact both target regions (0.002% of genomes; **Table 5**). Moreover, despite the presence of mutations in sequences flagged via *in-silico* analysis, follow-up *in-vitro* testing demonstrated no impact of these mutations on the ability of the QIAstat-Dx panels to detect all SARS-CoV-2 variants according to the nominal sensitivity specified in the instructions for use. Overall, these results lend further support to previous reports [5, 9] concerning the capacity of the SARS-CoV-2 panel when used on a QIAstat-Dx devices to detect the SARS-CoV-2 genome despite multiple detected mutations. Referring to previously published results [5, 9] and current results, it can be concluded that having an accurate PMS system allows for rapid detection of limitations of molecular tests such as QIAstat-Dx Panels. Such a system allows manufacturers to maintain good performance of any device, regardless of the pathogen targeted by the device.

### Importance of a PMS

Once an IVD assay is approved, the manufacturer is required to comply with PMS regulatory requirements, which include monitoring the performance of the test in the field and taking appropriate actions if an issue is identified. A primary component of PMS activities, in the context of infectious disease, is the requirement for IVD manufacturers to conduct regular assessment of the genetic variability of microorganisms targeted by their medical device(s). This required proactive approach is intended to ensure that genetic variability of the pathogen targeted by the device does not affect the sensitivity and specificity of the assay, thereby limiting the potential for incorrect results (i.e., false negatives and false positives) that could pose a risk to patient safety and public health [18]. The PMS systems are well defined for non-pandemic pathogens. In the case of SARS-CoV-2 that caused the COVID-19 outbreak, the first PMS requirements were not clearly specified. In the early phases of the pandemic, when the initial RT-PCR assays were developed to detect the presence of SARS-CoV-2 RNA in patient specimens, there were only a few genomes available on public databases. The general uncertainty about the extent to which genetic variability affected the detection of originally targeted regions of the SARS-CoV-2 genome led some manufacturers to strategically develop multiple RT-PCR assays, each targeting different regions of the viral genome, to reduce the risk of false-negative results—an approach that has demonstrated success since its implementation [5, 8]. When the current analysis was performed, and since the start of the COVID-19 pandemic, over 56,000 mutations (including polymorphisms, deletions, and insertions) had been recorded across the genome of SARS-CoV-2 among the 14 million genomes available in genomic databases [19]. This posed a clear challenge to diagnostic assay manufacturers who need to ensure their commercial assays can maintain originally established performance against the early SARS-CoV-2 strains.

Currently, there is a lack of minimum requirement or basic instruction set forth by regulators on a specific and necessary approach to assess the potential impact of genetic variability on device performance. That said, scientific reports have issued warnings on the extent to which genetic variability can affect commercial SARS-CoV-2 RT-PCR assays [20–22]. Previous efforts to establish protocols that examine the impact of genetic variability on assay performance have primarily concentrated on the *in-silico* assessment of how mutations affect specific assays, such as RT-PCR, and typically involve analyzing positional base changes and estimating the alterations in melting temperature that result from the corresponding genetic mismatches [19, 20]. Therefore, one of requirements should be that manufacturers have a well-established PMS for pathogen that led to pandemics such as SARS-CoV-2, which will allow regular and replicable monitoring of the pathogen’s genetic variability.

To our knowledge, this is the first study describing a detailed, generalizable three-step workflow that assesses the impact of a pathogen’s genetic variability on the sensitivity and specificity of an RT-PCR assay. Briefly, this three-step workflow encompasses a multifunctional bioinformatics pipeline *in-silico* assessment that includes a sequence-detection algorithm to facilitate subsequent *in-vitro* testing according to risk-based criteria. This generic workflow offers a practical and proactive approach to monitor and address the potential impact of genetic variability on the sensitivity of RT-PCR assays. By incorporating this novel workflow into the PMS activities of medical diagnostic devices, manufacturers can better anticipate and respond to emerging challenges posed by viral mutations, safeguarding patient safety and public health. Moreover, the successful implementation of this workflow could serve as a model for the development of future PMS strategies, fostering a more resilient and adaptive healthcare landscape in the face of evolving pathogens and future pandemics.

## Supporting information

S1 Table SARS-CoV-2 variants and associated WHO labels. (XLSX)

S2 Table Parameters of mutations associated with target genes. (XLSX)

S3 Table Study information for SARS-CoV-2, Flu A-B, RSV Panel. (XLSX)

S4 Table Sequence IDs and critical mutations in both targets. (XLSX)

S5 Table Data availability details. (PDF)
