# Supplementary material for: Monitoring SARS-CoV-2 genetic variability: A post-market surveillance workflow for combined bioinformatic and laboratory evaluation of commercial RT-PCR assay performance
Source: PLoS One. 2024 Jan 12;19(1):e0294271. doi: 10.1371/journal.pone.0294271 (PMC10786374; doi:10.1371/journal.pone.0294271)
Supplement: S5 Table — (PDF) [file pone.0294271.s005.pdf]

SUPPLEMENTAL TABLE

Data Availability

GISAID Identifier:	EPI_SET_230206vz
doi:	10.55876/gis8.230206vz

All genome sequences and associated metadata in this dataset are published in GISAID's EpiCoV database. To view the contributors of each individual sequence with details such as accession number, Virus name, Collection date, Originating Lab and Submitting Lab and the list of Authors, visit 10.55876/gis8.230206vz

Data Snapshot

- EPI_SET_230206vz is composed of 11,628,150 individual genome sequences.
- The collection dates range from 2010-12-06 to 2022-07-04;
- Data were collected in 220 countries and territories;
- All sequences in this dataset are compared relative to hCoV-19/Wuhan/WIV04/2019 (WIV04), the official reference sequence employed by GISAID (EPI_ISL_402124). Learn more at https://gisaid.org/WIV04.

Disclaimer: This supplementary material is hosted by Eurosurveillance as supporting information alongside the article "Monitoring SARS-CoV-2 genetic variability: A post-market surveillance workflow for combined bioinformatic and laboratory evaluation of commercial RT-PCR assay performance", on behalf of the authors, who remain responsible for the accuracy and appropriateness of the content. The same standards for ethics, copyright, attributions and permissions as for the article apply. Supplements are not edited by Eurosurveillance and the journal is not responsible for the maintenance of any links or email addresses provided therein.
